# Preparation and in vitro Immunoreactivity of *Rosa rugosa* Polysaccharide Liposomes

**DOI:** 10.5812/ijpr-161557

**Published:** 2025-08-25

**Authors:** Qinfang He, Riqing Cheng, Jiahao Shi, Ta Na, Qiherima Wang, Sarangowa Ochir

**Affiliations:** 1 Inner Mongolia Medical University, Hohhot, China

**Keywords:** Immunomodulation, *Rosa rugosa* Polysaccharides, Drug Delivery, Cytokine Secretion

## Abstract

**Background:**

* Rosa rugosa* polysaccharides (RRP), a principal active component derived from *R. rugosa* Thunb., exert immunomodulatory effects. However, their therapeutic application is limited by rapid metabolism, short duration of action, and low bioavailability.

**Objectives:**

To optimize the preparation process of *Rosa rugosa* polysaccharide liposomes (RRPL) and evaluate their immune cell activation in vitro.

**Methods:**

The RRPL were prepared using the reverse-phase evaporation method, with encapsulation efficiency (EE) as the primary evaluation criterion. An orthogonal test was employed to optimize the preparation parameters. Various characteristics of RRPL were assessed, including morphology, particle size, Polydispersity Index (PDI), zeta potential, cumulative release rate in vitro, and stability. The immunological effects of RRPL were evaluated through cellular assays involving mouse spleen lymphocytes, peritoneal macrophages, and bone marrow-derived dendritic cells (BMDCs).

**Results:**

The RRPL demonstrated an EE of approximately 81.96%, an average drug loading (DL) capacity of 13.86%, a particle size of 124.00 nm, a PDI of 0.23, and a zeta potential of -12.97 mV. The formulated RRPL exhibited high EE and DL capacity, alongside favorable slow-release properties and stability. These enhancements led to improved drug bioavailability and prolonged duration of action. Furthermore, RRPL significantly promoted the proliferation of spleen lymphocytes; enhanced the phagocytic activity of peritoneal macrophages; increased the secretion of interleukin (IL)-6, IL-1β, and interferon (IFN)-γ; activated immature BMDCs; and induced the maturation of BMDCs, resulting in increased production of IL-12p70 and tumor necrosis factor (TNF)-α.

**Conclusions:**

This study successfully developed RRPL that markedly enhance immune cell activation compared with RRP. These findings provide a theoretical foundation for further exploration and development of these liposomal formulations.

## 1. Background

The immune system is essential for protecting the body from pathogens and harmful substances; it comprises cells, tissues, and organs that work synergistically to maintain immune balance. Prolonged irregularities in diet, rest, sleep, fatigue, and nutritional deficiencies can lead to immune disorders, manifesting in conditions such as autoimmune diseases, allergies, and cancers (1, 2). Traditionally, immunomodulatory drugs are chemically synthesized, which often causes major side effects and leads to high production costs. In contrast, plant-derived immunomodulatory components effectively treat various diseases and mitigate the challenges associated with synthetic drugs (3). Numerous studies have highlighted that natural polysaccharides offer safety and low toxicity, making them suitable for immunomodulation in clinical settings (4, 5).

*Rosa rugosa*, a member of the Rosaceae family, is found in countries such as China, South Korea, and Japan and is used in both medicine and the food industry (6, 7). Its active constituents include polysaccharides, phenolic acids, flavonoids, and terpenoids (8, 9). Notably, *Rosa rugosa* polysaccharides (RRP) exert immunomodulatory, anticancer, hypoglycemic, and antioxidative effects (10-12). However, their rapid metabolism, short duration of action, and low bioavailability limit their clinical application.

The nano drug delivery system (NDDS) refers to the use of nanotechnology to disperse or encapsulate drugs in nanoscale carriers to form a drug delivery system with specific structure and function, including liposomes, nanoparticles, micelles, and so on. Among them, liposomes are one of the earliest FDA-approved nanocarriers for clinical use. Liposomes are small vesicles composed of lipid bilayers that exhibit enhanced biocompatibility, biodegradability, and low toxicity, making them one of the most prevalent nanodrug delivery systems (13-15). These structures can extend the biological half-life of drugs, improve the bioavailability and stability of drugs, and mitigate the toxic side effects of pharmaceuticals (16, 17). Moreover, liposomes act as immune adjuvants, effectively stimulating both innate and adaptive immune responses, promoting immune cell proliferation, and enhancing cytokine secretion (18-20).

Previous studies have demonstrated that the immunomodulatory activity of polysaccharides can be considerably enhanced when formulated into liposomes. For example, Bo et al. (21) prepared *Lycium barbarum* polysaccharide liposomes (LBPL), achieving an encapsulation efficiency (EE) of 86%. Compared with *L. barbarum* polysaccharides, LBPL markedly enhanced the proliferation of mouse dendritic precursor cells; promoted the expression levels of MHC-II, CD80, and CD86; and improved antigen presentation capabilities. Additionally, LBPL stimulated the secretion of interleukin (IL)-12 and tumor necrosis factor (TNF)-α while upregulating the expression levels of TLR4, MyD88, and TRAF6 as well as NF-κB mRNA and protein.

Liu et al. (22) developed *Rehmannia glutinosa* polysaccharide liposomes (RGPL). In vitro experiments indicated that at equivalent concentrations, macrophages treated with RGPL produced higher levels of IL-1β, interferon (IFN)-β, IL-6, IL-12, and TNF-α than those treated with *R. glutinosa* polysaccharides. Furthermore, the phagocytic capacity of RGPL was found to be superior to that of RGP in relation to macrophages.

## 2. Objectives

**Figure 1.**
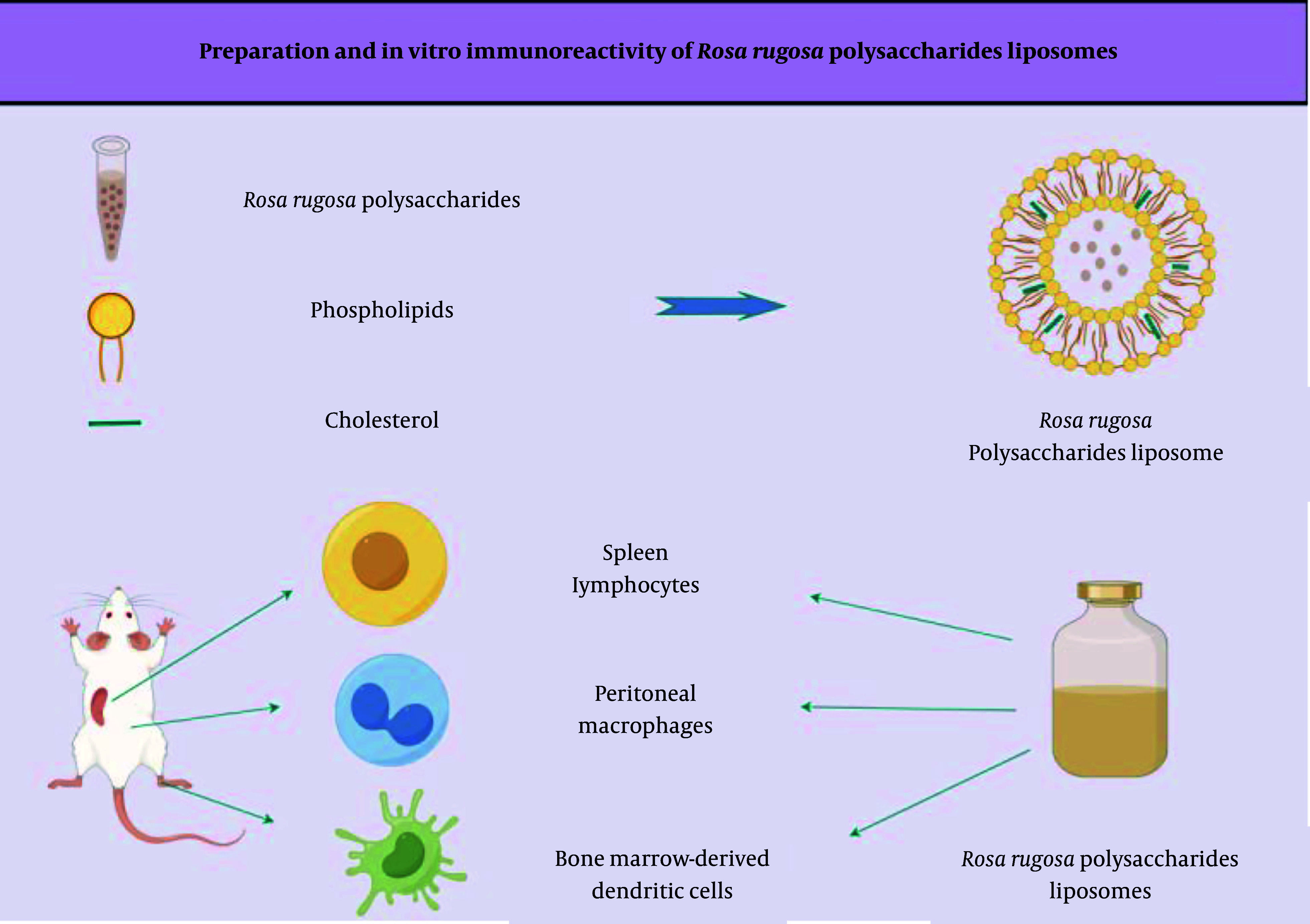
Schematic diagram of the preparation of RRPL and their in vitro immunological activity test using Figdraw. (Abbreviation: RRPL,* Rosa rugosa* polysaccharide liposomes).

In this study, RRP were used as a raw material, and liposomes served as a drug carrier for preparing *Rosa rugosa* polysaccharide liposomes (RRPL) via the reverse evaporation method. This approach aimed to improve the stability and bioavailability of RRP and evaluate their immune cell activation in vitro (schematic diagram shown in Figure 1. 

## 3. Methods

### 3.1. Materials and Animals

The RRP with a purity greater than 90% were obtained from Sichuan Vicqi Biotechnology Company, China. Phospholipids and cholesterol were purchased from China Ron Reagent Company, China. Phytohemagglutinin (PHA) was acquired from Aladdin, China, and lipopolysaccharide (LPS) from Yuan Ye, China. IL-6, IL-1β, IFN-γ, TNF-α, and IL-12 p70 kits were obtained from Yuan Mu, China. Recombinant mouse granulocyte-macrophage colony-stimulating factor (RMGM-CSF) and recombinant mouse IL-4 (RMIL-4) were purchased from Sino Biological, China. Additionally, 4-week-old male Institute of Cancer Research (ICR) mice, weighing 18 - 25 g, were obtained from the Laboratory Animal Center of Inner Mongolia Medical University. The housing conditions included a controlled temperature of 22°C ± 2°C and humidity of 55% ± 5%.

### 3.2. Preparation and Characterization of Rosa rugosa Polysaccharide Liposomes

#### 3.2.1. Preparation of Rosa rugosa Polysaccharide Liposomes

The RRPL were prepared using the reverse-phase evaporation method (23). Precise amounts of phospholipids and cholesterol were weighed and dissolved in anhydrous ether along with the RRP solution. The mixture was subjected to ultrasonication using an ultrasonic cell disruptor. Subsequently, the mixture was transferred to an eggplant-shaped flask and placed on crushed ceramic tiles. The solution was subjected to downward pressure treatment using a rotary evaporator at 30°C in a water bath. Phosphate-buffered saline (PBS) was added to facilitate mixing, and the mixture was subjected to continued rotary evaporation under atmospheric pressure. Hydration was performed at 30°C for 2 hours in the rotary evaporator. Finally, ultrasonic homogenization was stopped, and the suspension was filtered through a 0.22-μm microporous membrane, resulting in the formation of an RRPL suspension.

Encapsulation efficiency and drug loading (DL) capacity were determined using ultracentrifugation and ultraviolet spectrophotometry (24). Encapsulation efficiency was calculated using the formula: EE% = (WT - WF)/WT × 100%, where WT represents the total amount of drug and WF denotes the amount of free drug. Drug loading was calculated using the formula: DL% = (WT - WF)/WP, where WP is the total amount of phospholipids and cholesterol.

#### 3.2.2. Optimization of the Preparation Process of Rosa rugosa Polysaccharide Liposomes

Liposomes were prepared according to the procedure outlined in section 3.2.1. The impact of four variables — phospholipid-to-cholesterol mass ratio, drug concentration, ultrasonic time, and water bath temperature — on the EE of liposomes was evaluated individually. According to the results, (A) phospholipid-to-cholesterol mass ratio; (B) ultrasonic time; and (C) drug concentration were identified as the main factors influencing the RRPL coating rate. Encapsulation efficiency was used as an Evaluation Index, and L9 (3^3^) orthogonal experiments were conducted to optimize the preparation conditions for RRPL.

#### 3.2.3. Characterization of Rosa rugosa Polysaccharide Liposomes

The RRPL morphology was examined using transmission electron microscopy (G2 F20, FEI Tecnai, USA). Additionally, the particle size, Polydispersity Index (PDI), and zeta potential (n = 3) of RRPL were assessed using a nanometer particle size analyzer (SZ-100V2, HORIBA, China). In vitro drug release tests for RRPL and RRP were conducted using the dialysis method. The PBS was employed as the release medium. Specifically, 5 mL of RRPL and RRP were placed in a dialysis bag, which was then securely sealed at both ends with cotton thread. The dialysis bag was submerged in a beaker containing 50 mL of PBS and positioned in a constant-temperature shaker set at 37°C and 100 rpm for the duration of the release study. Samples were collected at 0.5-, 1-, 2-, 3-, 4-, 6-, 8-, 10-, 12-, and 24-hour intervals. To maintain a consistent release environment, equal volumes of pre-warmed PBS were added to the beaker after each sample withdrawal. The in vitro release rate was determined using the phenol-sulfuric acid method, and a cumulative release rate curve was plotted based on these measurements. Additionally, RRPL were stored in a refrigerator at 4°C, and their particle size, PDI, and zeta potential were measured on days 0, 7, 14, 21, 28, 35, and 42 to assess their stability.

### 3.3. Effect of Rosa rugosa Polysaccharide Liposomes on the Immune Function of Mouse Spleen Lymphocytes

#### 3.3.1. Preparation of Mouse Spleen Lymphocytes

Mouse spleen lymphocytes were collected from ICR mice using a modified protocol (25). Under aseptic conditions, the spleen was excised and placed on a 100-mesh cell sieve. It was gently ground with a sterile syringe and rinsed with PBS, followed by pipetting to obtain a uniform suspension. The resulting suspension was transferred to 15-mL centrifuge tubes and centrifuged at 1500 rpm for 8 minutes. The supernatant was discarded, and an erythrocyte lysate was added before performing a second centrifugation at the same speed and duration. After removing the supernatant, the pellet was washed twice with PBS. The cell suspension was then diluted to approximately 2.5 × 10^6^ cells/mL with RPMI-1640 complete culture medium.

#### 3.3.2. Cytotoxicity Assay

The spleen lymphocyte suspension prepared above was aliquoted into a 96-well plate, with 100 μL of RRPL and RRP solutions at different concentrations added to the wells, including a cell control (CC) group. After incubation at 37°C for 44 hours under a 5% CO_2_ atmosphere, 30 μL of 3-(4,5-dimethylthiazol-2-yl)-2,5-diphenyltetrazolium bromide (MTT) solution was added to each well and incubated for an additional 4 hours. The supernatant was then removed, and 100 μL of dimethyl sulfoxide (DMSO) was added to each well. The absorbance at 570 nm was measured using a microliter enzyme-linked immunosorbent assay (ELISA; Spectramax i3, Molecular Devices, China) to evaluate cell viability.

#### 3.3.3. Cell Proliferation Assay

A spleen lymphocyte suspension was added to 96-well plates at a volume of 80 μL per well. Following this, LPS (5 μg/mL), PHA (10 μg/mL), and RPMI-1640 medium were added to each well at a volume of 20 μL. Various concentrations of RRPL and RRP (ranging from 105 to 6.563 μg/mL), along with blank liposomes (BL), were also introduced, resulting in a total volume of 100 μL per well, which included control wells for LPS/PHA and CC. After incubation, 30 μL of MTT solution was added to each well and allowed to incubate for an additional 4 hours. Following this period, the supernatant was removed, and 100 μL of DMSO was added to each well to dissolve formazan crystals. The absorbance was then measured at 570 nm using a microliter ELISA to assess cell viability.

### 3.4. Effect of Rosa rugosa Polysaccharide Liposomes on the Immune Function of Mouse Peritoneal Macrophages

#### 3.4.1. Preparation of Mouse Peritoneal Macrophages

Mouse peritoneal macrophages were isolated using a modified method (26). Mice were intraperitoneally injected with a 6% starch broth. Two days later, 5 mL of PBS was injected into the abdominal cavity, and the abdomen was gently rubbed for 5 minutes. The abdominal fluid was then aspirated into a centrifuge tube with a 2.5-mL syringe. The sample was centrifuged at 1500 rpm for 5 minutes, and the supernatant was discarded. The pellet was washed with PBS to obtain the peritoneal macrophage suspension. Finally, Dulbecco’s modified Eagle medium was added to adjust the cell density to 5.0 × 10^5^ cells/mL.

#### 3.4.2. Phagocytic Activity of Peritoneal Macrophage Assay

The peritoneal macrophage suspension obtained above was plated at a volume of 3 mL per well in 6-well plates and incubated for 4 hours to allow cell adhesion. Following adherence, the supernatant was removed, and RRPL, RRP, and BL were added to separate wells. Cell control and LPS-positive control groups were also prepared at a volume of 3 mL per well. There were four wells per group. After the plates were incubated for 48 hours, the supernatant was discarded, and peritoneal macrophages were washed twice with PBS. The Vybrant^™^ Phagocytosis Assay kit instructions were followed.

#### 3.4.3. Measurement of Interleukin-6, Interleukin-1β, and Interferon-γ Levels in Peritoneal Macrophages

Peritoneal macrophages were plated onto 96-well plates. After incubation for 4 hours to allow cell adhesion, the supernatant was removed. The RRPL, RRP, and BL at concentrations of 105, 52.5, and 26.25 μg/mL, respectively, were added to the wells at a volume of 100 μL per well. Concurrently, CC and LPS-positive control groups were established. The plates were incubated for 24 hours, after which the supernatant was collected. The concentrations of IL-6, IL-1β, and IFN-γ were measured using an ELISA kit according to the manufacturer’s instructions.

### 3.5. Effect of Rosa rugosa Polysaccharide Liposomes on the Immune Function of Mouse Bone Marrow-Derived Dendritic Cells

#### 3.5.1. Preparation of Mouse Bone Marrow-Derived Dendritic Cells

Bone marrow-derived dendritic cells (BMDCs) were isolated according to a previous protocol (21) with minor modifications. Under aseptic conditions, mouse femurs and tibias were dissected and their ends were removed. RPMI-1640 medium was aspirated into a syringe and used to flush bone marrow cavities to collect BMDCs. These cells were centrifuged at 1500 rpm for 8 minutes, and the supernatant was discarded. An erythrocyte lysis solution was then added, and the mixture was incubated for 2 minutes at 25°C. RPMI-1640 medium was added to halt the lysis process. Next, the suspension was centrifuged at 1500 rpm for 8 minutes, the supernatant was discarded, and the cells were washed with PBS. The final cell density was adjusted to 2.0 × 10^6^ cells/mL using the RPMI-1640 complete medium.

#### 3.5.2. Mouse Dendritic Cell Precursor Cell Proliferation Assay

The cells prepared above were seeded into 96-well plates at a volume of 100 μL per well. After 15 hours of incubation, RRPL, RRP, and BL were added to the wells at concentrations of 105, 52.5, and 26.25 μg/mL, respectively, at a volume of 100 μL per well. A CC group was established concurrently. After 48 hours of incubation, the cells were treated with 30 μL of MTT per well and further incubated at 37°C for 4 hours. Subsequently, the supernatant was discarded, and 100 μL of DMSO was added to each well. The absorbance at 570 nm was measured using a microplate reader.

#### 3.5.3. Stimulation of Bone Marrow-Derived Dendritic Cells

The cell density was adjusted to 2.0 × 10^6^ cells/mL, and the cells were seeded into a 6-well plate for cultivation. After 24 hours, the supernatant was discarded, and RPMI-1640 complete medium, supplemented with RMGM-CSF and RMIL-4, was added. The medium was replaced with fresh medium on the 3rd and 5th days. By the 7th day of culture, the desired mature BMDCs were obtained, and their morphology was monitored throughout the cultivation period.

#### 3.5.4. Measurement of Interleukin-12p70 and Interferon-α Levels in Bone Marrow-Derived Dendritic Cells

Mature BMDCs were adjusted to a concentration of 5.0 × 10^5^ cells/mL and plated in a 96-well plate. The RRPL, RRP, and BL were prepared at concentrations of 105, 52.5, and 26.25 μg/mL, respectively. The plate was incubated for 24 hours. Following incubation, the supernatant was collected, and IL-12p70 and TNF-α levels were measured using an ELISA kit.

### 3.6. Statistical Analysis

The data are presented as mean ± standard error of the mean. Statistical analysis was performed using IBM SPSS Statistics 26 software, including analysis of variance (ANOVA) and post-hoc multiple comparisons. A P-value of < 0.05 was considered to indicate statistical significance.

## 4. Results

### 4.1. Optimization of the Preparation Process of Rosa rugosa Polysaccharide Liposomes

#### 4.1.1. Effects of Individual Factors on the Encapsulation Efficiency of Rosa rugosa Polysaccharide Liposomes

**Figure 2.**
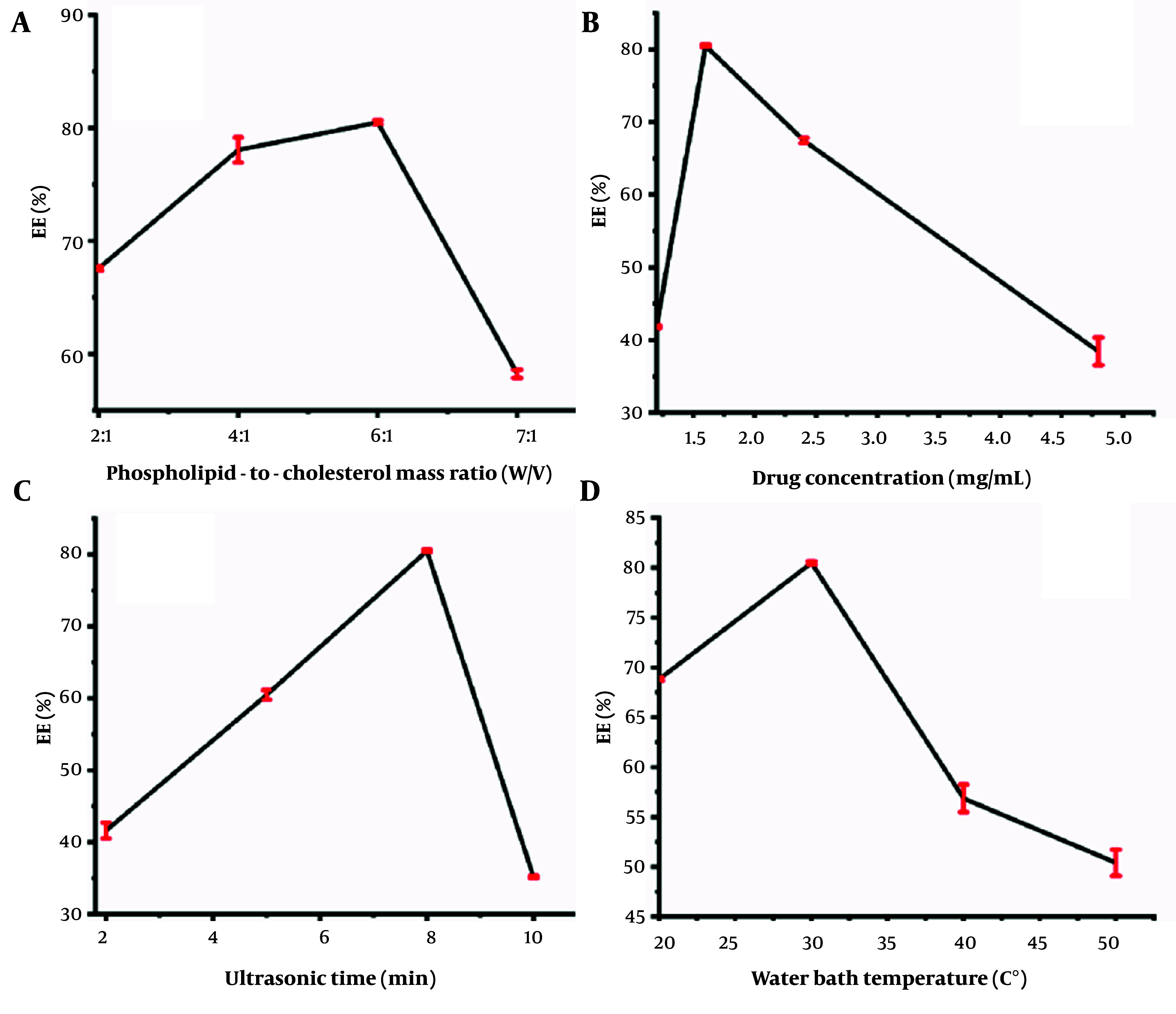
Effects of individual factors on the EE of RRPL (n = 3). A, phospholipid-to-cholesterol mass ratio; B, drug concentration; C, ultrasonic time; and D, water bath temperature. (Abbreviations: EE, encapsulation efficiency; RRPL, *Rosa rugosa* polysaccharides liposomes).

The effects of the phospholipid-to-cholesterol mass ratio, drug (RRP) concentration, ultrasonic time, and water bath temperature on the EE of RRPL are shown in Figure 2A - D. The phospholipid-to-cholesterol mass ratios were varied at 2:1, 4:1, 6:1, and 7:1 for preparing RRPL, with all other conditions remaining constant. As shown in Figure 2A, the EE of RRPL was highest at a phospholipid-to-cholesterol mass ratio of 6:1.

The drug concentrations were varied at 4.8, 2.4, 1.6, and 1.2 mg/mL to prepare RRPL, with all other conditions remaining constant. As shown in Figure 2, the highest encapsulation rate of RRPL (80.50%) was achieved at a drug concentration of 1.6 mg/mL. The ultrasonic times were varied at 2, 5, 8, and 10 minutes for preparing RRPL, with all other conditions remaining constant. As shown in Figure 2C, the EE of RRPL peaked at an ultrasonic time of 8 minutes. The water bath temperatures were varied at 20°C, 30°C, 40°C, and 50°C for preparing RRPL, with all other conditions remaining constant. As presented in Figure 2D, the highest EE of RRPL was achieved at a water bath temperature of 30°C. Therefore, the EE of RRPL was considerably influenced by the phospholipid-to-cholesterol ratio, drug concentration, ultrasonic time, and water bath temperature.

#### 4.1.2. Orthogonal Test

The phospholipid-to-cholesterol mass ratio (A), drug concentration (B), and ultrasonic time (C) were used as the main factors for the orthogonal test to optimize the preparation conditions for RRPL. In this test, the polar deviation (R) indicates the degree of influence of each factor on the encapsulation rate, with a larger R value reflecting a greater impact. As detailed in Table 1, the influence of these factors follows the order: Drug concentration (B) > phospholipid-to-cholesterol mass ratio (A) > ultrasonic time (C). Based on the K-values, the optimal formulation for the preparation of RRPL was determined to be B2A3C3, corresponding to a phospholipid-to-cholesterol mass ratio of 6:1, a drug concentration of 1.6 mg/mL, and an ultrasonic time of 8 minutes.

**Table 1.** Analysis of L9(3^3^) Orthogonal Test on Preparation of *Rosa rugosa* Polysaccharide Liposomes ^a, b, c^

No.	Factors			Determination Index
	A	B	C	EE (%)
**1**	2:1	2.4	2	47.68 ± 0.54
**2**	2:1	1.6	5	58.64 ± 0.49
**3**	2:1	1.2	8	51.54 ± 0.66
**4**	4:1	2.4	8	47.22 ± 0.23
**5**	4:1	1.6	2	62.08 ± 0.60
**6**	4:1	1.2	5	58.51 ± 1.08
**7**	6:1	2.4	5	51.62 ± 0.77
**8**	6:1	1.6	8	82.03 ± 0.67
**9**	6:1	1.2	2	57.56 ± 0.70
**K**_**1**_	52.620	48.840	55.773	-
**K**_**2**_	50.005	67.583	51.341	-
**K**_**3**_	64.752	50.954	60.273	-
**R**	14.747	18.743	8.922	-

Abbreviation: EE, encapsulation efficiency.

^a^ K-values represents level mean.

^b^ R-values represents range.

^c^ The data is expressed as the man ± SEM (n = 3).

Table 2 presents the ANOVA results. There was a significant difference in the effect of drug concentration on the EE of RRPL (P < 0.05), whereas there were no significant differences in the effects of the phospholipid-to-cholesterol mass ratio and ultrasonic time on the EE of RRPL (P > 0.05), indicating that drug concentration is the most important factor in the preparation of RRPL.

**Table 2.** Analysis of Variance on L9(3^3^) Orthogonal Test on Preparation of *Rosa rugosa* Polysaccharide Liposomes

Factor	Sum of Square of Deviation	Deviation	F	P-Value
**A**	297.484	2	11.750	0.078
**B**	611.243	2	24.143	0.040 ^a^
**C**	101.538	2	4.012	0.200
**Error**	25.318	2		

^a^ A P-value of < 0.05 is considered statistically significant.

#### 4.1.3. Characterization of Rosa rugosa Polysaccharide Liposomes

The average EE of RRPL was 81.96% ± 1.77% (n = 3), and the average DL capacity was 13.86% ± 0.35% (n = 3), indicating that the preparation process exhibits good repeatability, along with high EE and DL. Furthermore, the morphology, particle size, PDI, zeta potential, in vitro drug release, and stability of RRPL were evaluated.

**Figure 3.**
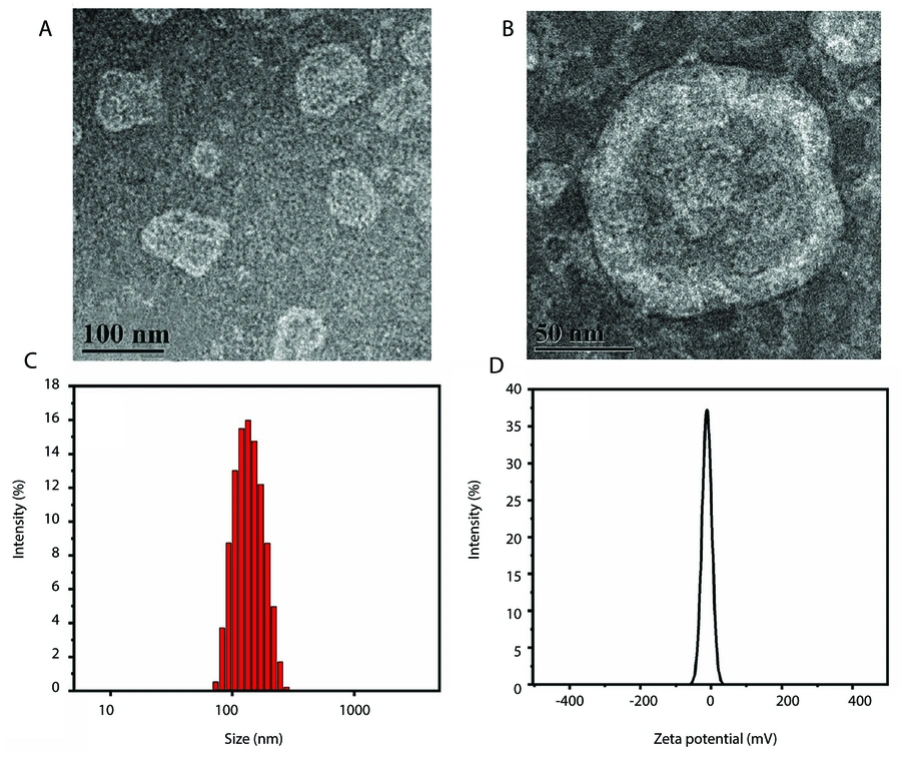
A, TEM images of RRPL (100 nm); B, TEM images of RRPL (50 nm); C, particle size distribution of RRPL; and D, zeta potential of RRPL. (Abbreviation: RRPL, *Rosa rugosa* polysaccharides liposomes).

Based on transmission electron microscopy (Figure 3A and B), the morphology of RRPL prepared using the optimal formulation was found to be subspherical without any signs of agglomeration. Additionally, the particle size and PDI of RRPL were measured as 124.00 ± 3.28 nm and 0.230 ± 0.069 (Figure 3C), respectively, consistent with the results of transmission electron microscopy. Furthermore, the zeta potential of RRPL was determined to be -12.97 ± 1.63 mV (Figure 3D). 

**Figure 4.**
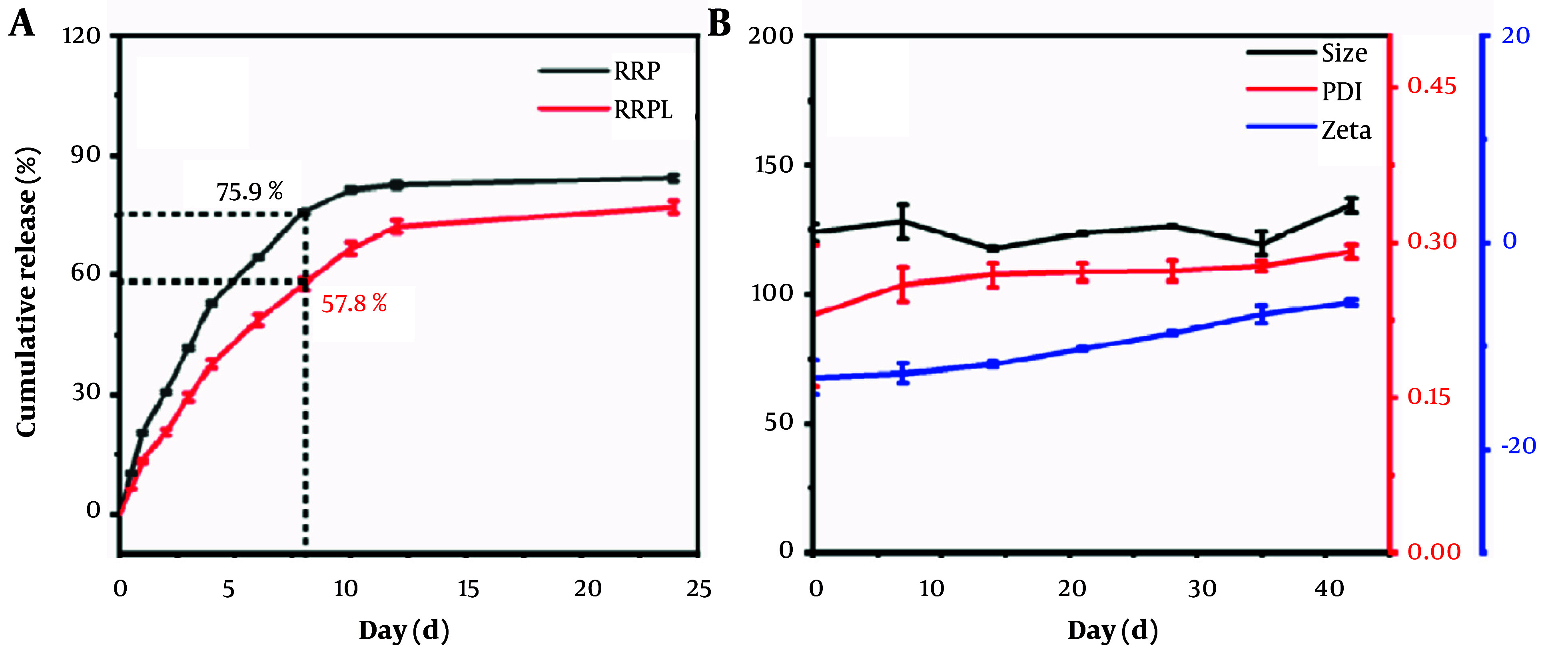
A, in vitro cumulative release curves of RRPL and RRP (n = 3); and B, stability of RRPL (n = 3). (Abbreviations: RRPL, *Rosa rugosa* polysaccharides liposomes; RRP, *R. rugosa* polysaccharides).

As illustrated in Figure 4A, the cumulative release rate of RRP reached 75.94% at 8 hours and 81.39% at 10 hours. In comparison, RRPL exhibited cumulative release rates of 57.80% and 66.74% at 8 and 10 hours, respectively, reaching 77.06% at 24 hours. These results indicate that RRPL demonstrate a notably slower release effect compared with RRP.

As shown in Figure 4B, the average particle size of RRPL at 4°C was measured as 124 nm (n = 3) on day 0, with a PDI of 0.23 (n = 3) and a zeta potential of -12.97 mV (n = 3). By day 42, the average particle size increased to 134.67 nm, the PDI rose to 0.29, and the zeta potential decreased to -5.70 mV. Specifically, the particle size increased by approximately 10.67 nm, the PDI increased by approximately 0.06, and the zeta potential decreased by approximately 7.27 mV. These findings indicate minimal alterations in the particle size, PDI, and zeta potential of RRPL over a 42-day period, suggesting that RRPL demonstrate good stability when stored at 4°C.

### 4.2. Proliferation Effects of Rosa rugosa Polysaccharide Liposomes on Mouse Spleen Lymphocytes

#### 4.2.1. Cytotoxicity Assay

**Figure 5.**
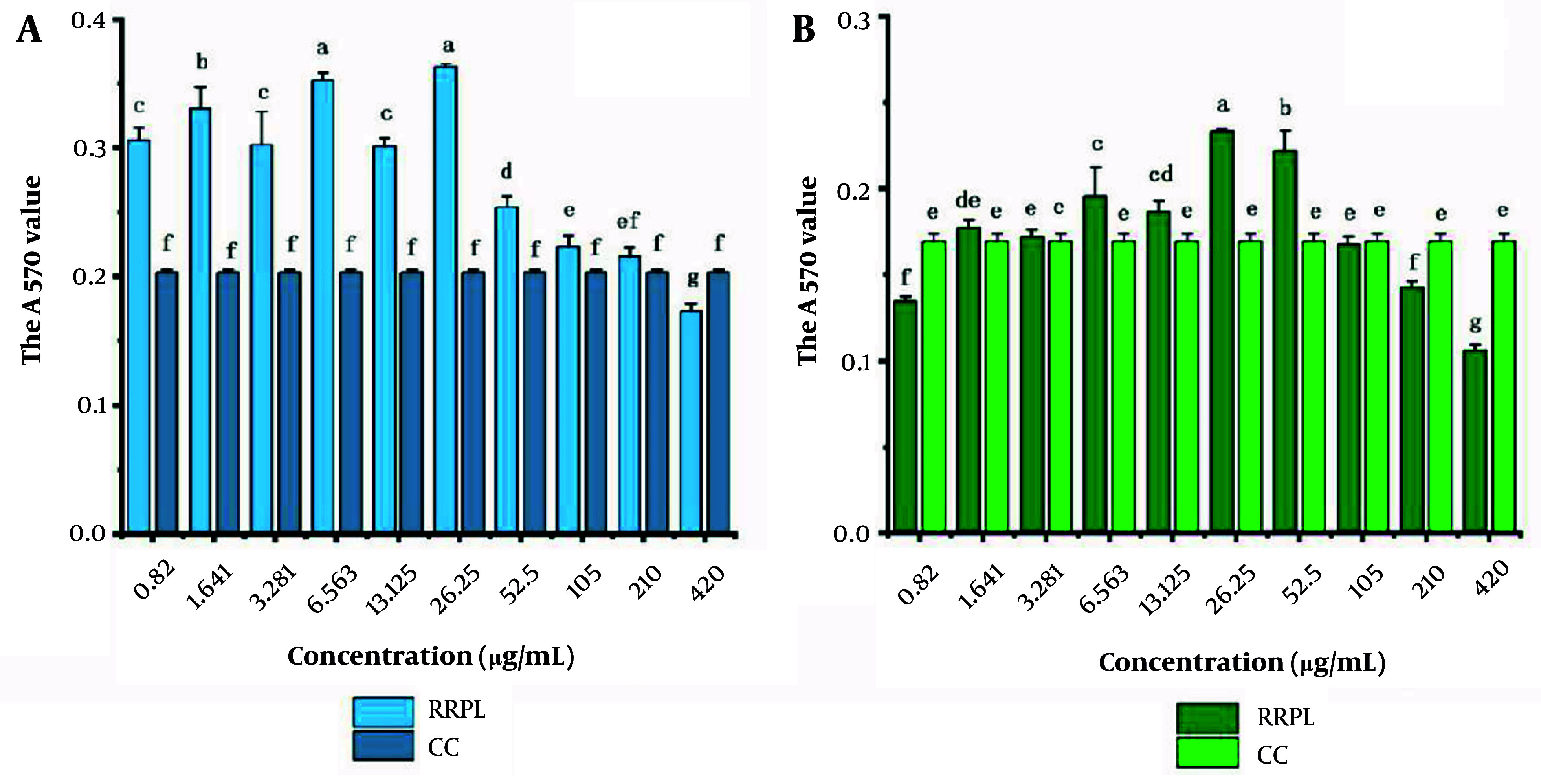
The cytotoxicity of RRPL and RRP solutions at different concentrations (0.82 - 420 μg/mL) against mouse spleen lymphocytes (n = 4). A, RRPL; B, RRP. The presence of identical letters in superscript a-f indicates no significant difference (P > 0.05), and the presence of different letters in superscript a-f indicates a significant difference (P < 0.05). (Abbreviations: RRPL, *Rosa rugosa* polysaccharides liposomes; RRP, *R. rugosa* polysaccharides; CC, cell control).

The cytotoxicity of RRPL and RRP solutions at different concentrations (0.82 - 420 μg/mL) against mouse spleen lymphocytes was assessed. As shown in Figure 5, there was no statistically significant difference in absorbance values between the CC group and the RRPL group at a concentration of 210 μg/mL (P > 0.05). However, the absorbance values of the RRPL group at other concentrations differed significantly compared with those of the CC group, indicating that RRPL significantly promote spleen lymphocyte proliferation within the concentrations of 0.82 - 105 μg/mL.

Similarly, no significant difference in absorbance values was observed between the CC group and the RRP group at a concentration of 105 μg/mL; however, the absorbance values of the RRP group at concentrations of 6.563 - 52.5 μg/mL were significantly higher compared with those of the CC group, suggesting that RRP also significantly enhance spleen lymphocyte proliferation within the concentrations of 6.563 - 52.5 μg/mL.

Based on these findings, the concentrations of 210 and 105 μg/mL were established as the maximum safe concentrations of RRPL and RRP, respectively, for promoting spleen lymphocyte proliferation.

#### 4.2.2. Proliferation Effects of Rosa rugosa Polysaccharide Liposomes on B and T Spleen Lymphocytes

**Figure 6.**
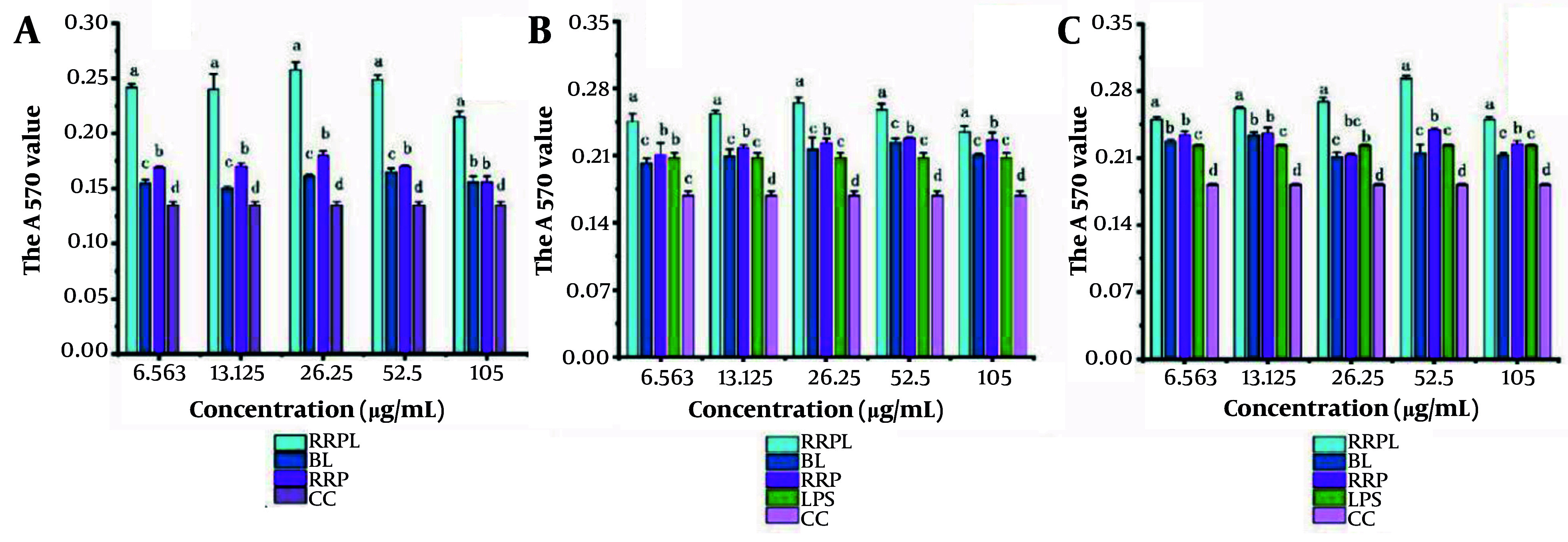
The proliferation effects of RRPL, BL, RRP, and CC at different concentrations (6.563 - 105 μg/mL) on spleen lymphocytes (n = 4). A, effect of single dose on spleen lymphocytes; B, effects of drugs combined with LPS on spleen lymphocytes; and C, effect of PHA combination on spleen lymphocytes. The presence of identical letters in superscript a-d indicates no significant difference (P > 0.05) and the different letters in superscript a-d indicates a significant difference (P < 0.05). (Abbreviations: RRPL, *Rosa rugosa* polysaccharides liposomes; RRP, *R. rugosa* polysaccharides; BL, blank liposomes; CC, cell control; LPS, lipopolysaccharide; PHA, Phytohemagglutinin).

The proliferation effects of RRPL, BL, RRP, and CC at concentrations of 6.563, 13.125, 26.25, 52.5, and 105 μg/mL on spleen lymphocytes are shown in Figure 6A. At all concentrations, the absorbance values of the RRPL group were significantly higher than those of the BL, RRP, and CC groups (P < 0.05). Additionally, within the 6.563 - 52.5 μg/mL concentration range, the absorbance values of the RRP group were significantly higher than those of the BL and CC groups. These results indicate that both RRPL and RRP stimulate the proliferation of spleen lymphocytes, with RRPL exhibiting a considerably stronger effect than RRP.

The experimental results shown in Figure 6B and C illustrate the proliferation effects of RRPL, BL, RRP, and CC on spleen lymphocytes when combined with LPS/PHA. Within the 6.563 - 105 μg/mL concentration range, the absorbance values at 570 nm of the RRPL group were significantly higher than those of the BL, RRP, LPS-positive control, and CC groups (P < 0.05). These findings indicate that RRPL significantly promote spleen lymphocyte proliferation, regardless of whether they are synergized with LPS or PHA, and exhibit a stronger effect than RRP.

### 4.3. Immunological Effects of Rosa rugosa Polysaccharide Liposomes on Mouse Peritoneal Macrophages

#### 4.3.1. Rosa rugosa Polysaccharide Liposomes Enhance the Phagocytic Activity of Peritoneal Macrophages

**Figure 7.**
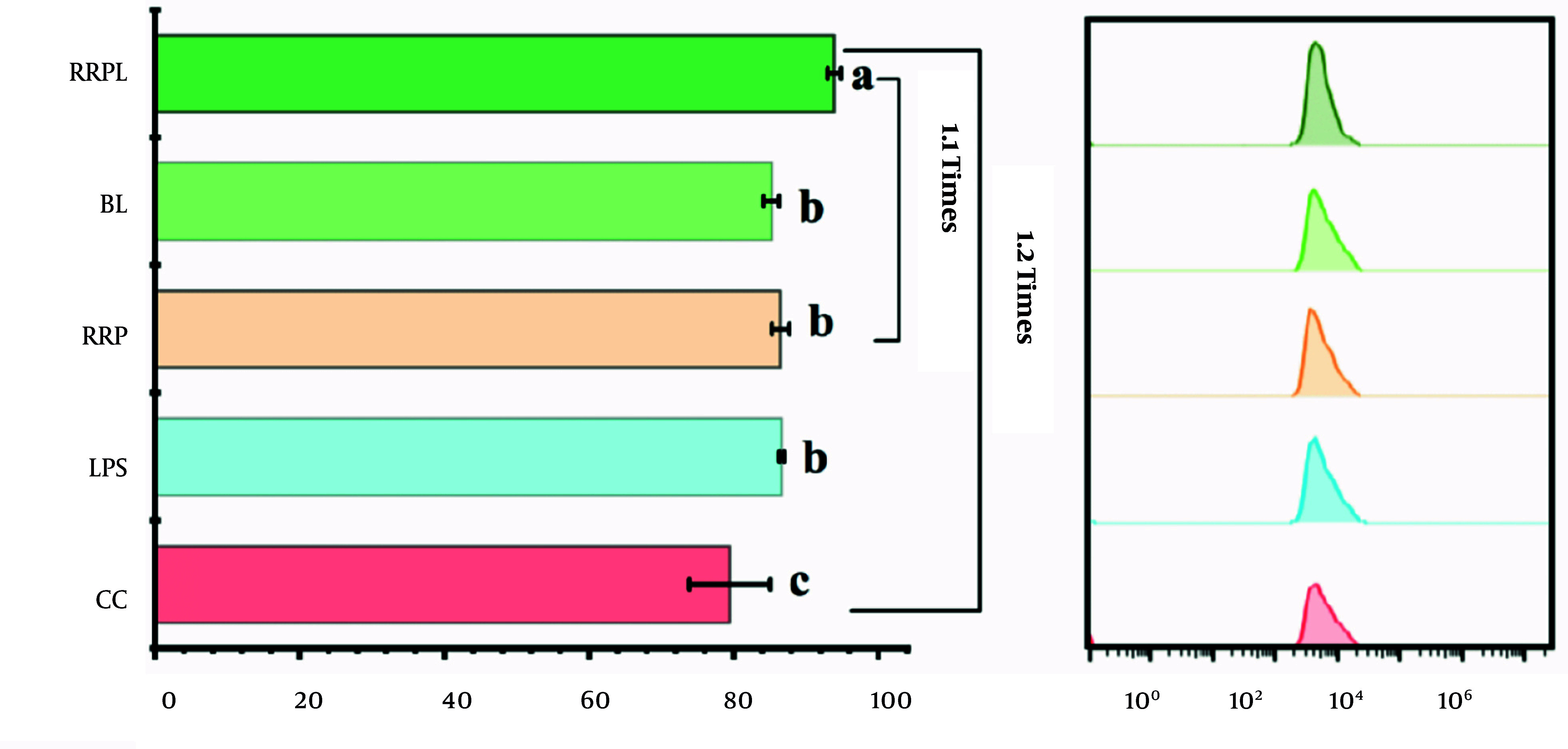
FACS histogram shows the phagocytic capacity of macrophages to phagocytic fluorescently labeled *Escherichia coli* (n = 4). The presence of identical letters in superscript a-c indicates no significant difference (P > 0.05) and the different letters in superscript a-c indicates a significant difference (P < 0.05).

The phagocytic ability of macrophages against fluorescently labeled *Escherichia coli* was assessed and expressed as a percentage of positive intracellular fluorescence. The results are presented in Figure 7. Peritoneal macrophages stimulated in the RRPL group exhibited a significantly higher percentage of positive fluorescence (93.9%) than those stimulated in the other groups (P < 0.05). The positive fluorescence percentage of the CC group was 80%. Therefore, the phagocytic capability of peritoneal macrophages stimulated in the RRPL group increased by approximately 20% compared with that in the CC group and by approximately 10% compared with that in the RRP group.

##### 4.3.2 Effects of Rosa rugosa Polysaccharide Liposome on Interleukin-6, Interleukin-1β , and Interferon-γ Secretion from Peritoneal Macrophages

**Figure 8.**
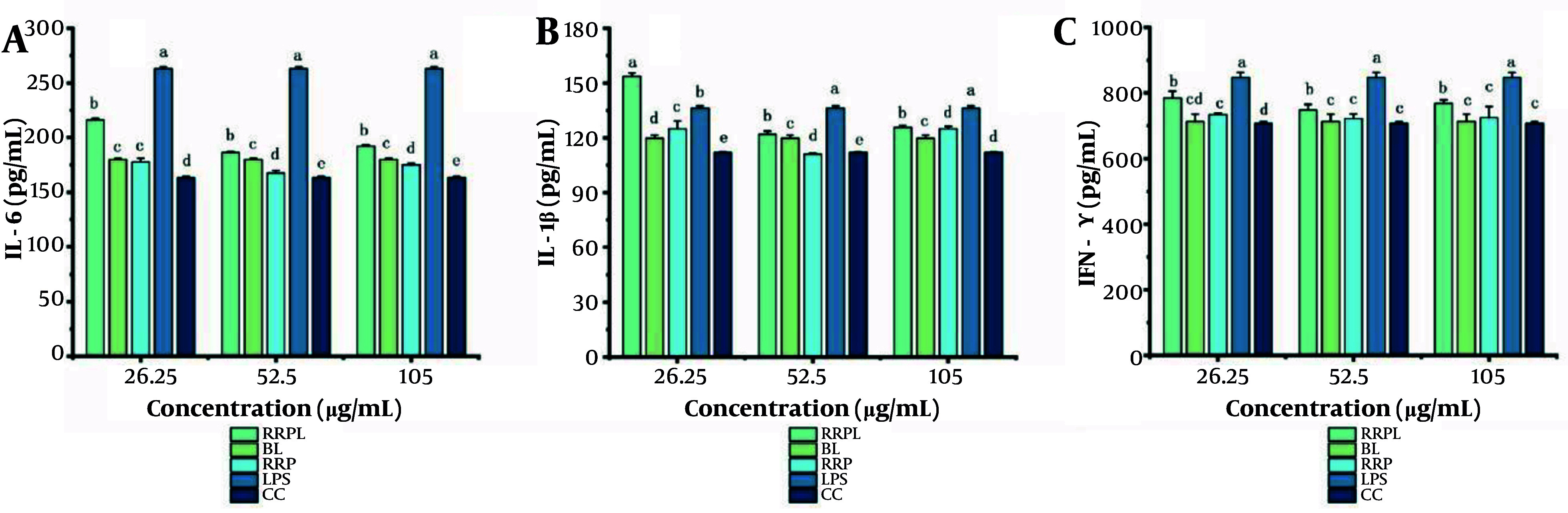
Effect of RRPL on the secretion of IL-6, IL-1β, and IFN-γ in macrophages (n = 4); the presence of identical letters in superscript a-e indicates no significant difference (P > 0.05) and the different lettters in superscrip a-e indicates a significant difference (P < 0.05). (Abbreviations: RRPL, *Rosa rugosa* polysaccharides liposomes; RRP, *R. rugosa* polysaccharides; BL, blank liposomes; CC, cell control; LPS, lipopolysaccharide; IL-6, interleukin-6; IL-1β, interleukin-1β; IFN-γ, interferon-γ).

The effects of RRPL on IL-6, IL-1β, and IFN-γ secretion from peritoneal macrophages were assessed via ELISA. As shown in Figure 8A, the LPS-positive control group at concentrations of 26.25, 52.5, and 105 μg/mL exhibited significantly higher levels of IL-6 than the other groups (P < 0.05). Similarly, the RRPL group demonstrated significantly higher IL-6 secretion than the other groups, except for the LPS-positive control group. These findings indicate that RRPL effectively promote IL-6 secretion from peritoneal macrophages compared with RRP.

The IL-1β assay results are depicted in Figure 8B. The RRPL group at a concentration of 26.25 μg/mL showed significantly higher IL-1β secretion than the other groups. Moreover, the LPS-positive control group at concentrations of 52.5 and 105 μg/mL exhibited significantly higher IL-1β levels than the other groups. However, the RRPL group at similar concentrations also demonstrated enhanced activity compared with the other groups, except for the LPS-positive control group.

As shown in Figure 8C, the LPS-positive control group at concentrations of 26.25, 52.5, and 105 μg/mL demonstrated significantly higher IFN-γ levels compared with the other groups. Additionally, the RRPL group at similar concentrations exhibited significantly higher activity than the other groups. These findings suggest that RRPL effectively promote IL-6, IL-1β, and IFN-γ secretion from peritoneal macrophages compared with RRP.

### 4.4. Immunological Effects of Rosa rugosa Polysaccharide Liposomes on Mouse Bone Marrow-Derived Dendritic Cells

#### 4.4.1. Proliferation Effects of Rosa rugosa Polysaccharide Liposomes on Mouse Bone Marrow-Derived Dendritic Cells

**Figure 9.**
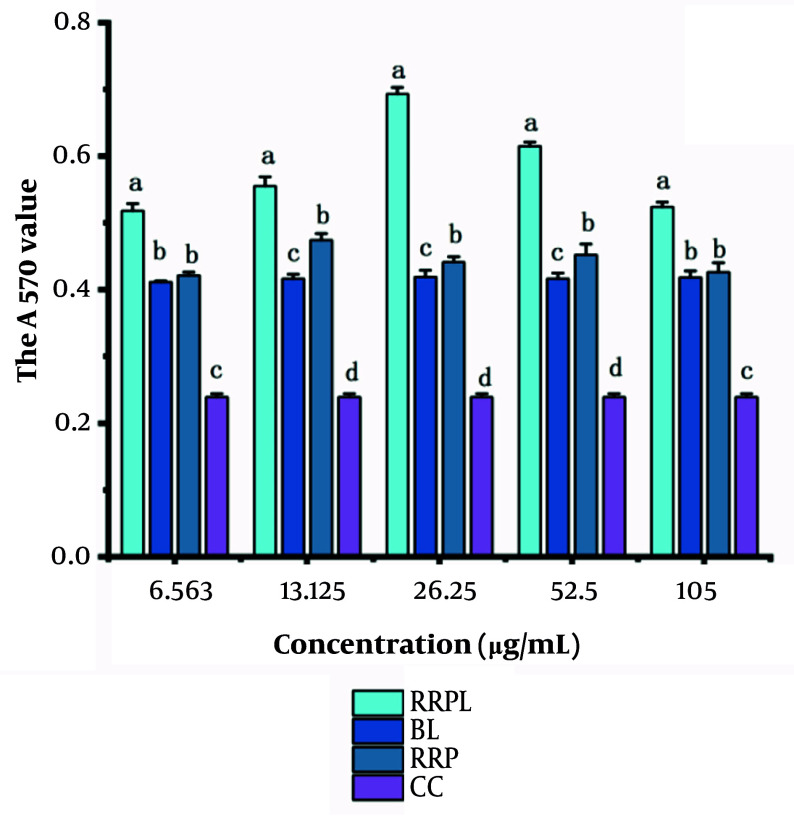
Proliferation effects of drugs on DC precursor cells (n = 4); the presence of identical letters in superscript a-d indicates no significant difference (P > 0.05) and the different letters in superscript a-d indicates a significant difference (P < 0.05). (Abbreviations: RRPL, *Rosa rugosa* polysaccharides liposomes; RRP, *R. rugosa* polysaccharides; BL, blank liposomes; CC, cell control).

The proliferation effects of RRPL, BL, RRP, and CC at concentrations of 6.563, 13.125, 26.25, 52.5, and 10^5^ μg/mL on dendritic cell (DC) precursor cells are shown in Figure 9. The RRPL group demonstrated significantly stronger effects compared with the other groups (P < 0.05). The absorbance values at 570 nm were highest at 26.25 μg/mL and lowest at 6.563 μg/mL for RRPL, indicating their potent ability to promote DC precursor cell proliferation at 26.25 μg/mL and reduced efficacy at 6.563 μg/mL. In contrast, the RRP group exhibited significantly stronger proliferation effects than all other groups, except for the RRPL group within the concentration range of 13.125 - 52.5 μg/mL. These results suggest that both RRPL and RRP promote DC precursor cell proliferation, with RRPL having a more pronounced effect.

#### 4.4.2. Effect of Rosa rugosa Polysaccharide Liposomes on Interleukin-12p70 and Tumor Necrosis Factor-α Secretion from Induced Mouse Bone Marrow-Derived Dendritic Cells

**Figure 10.**
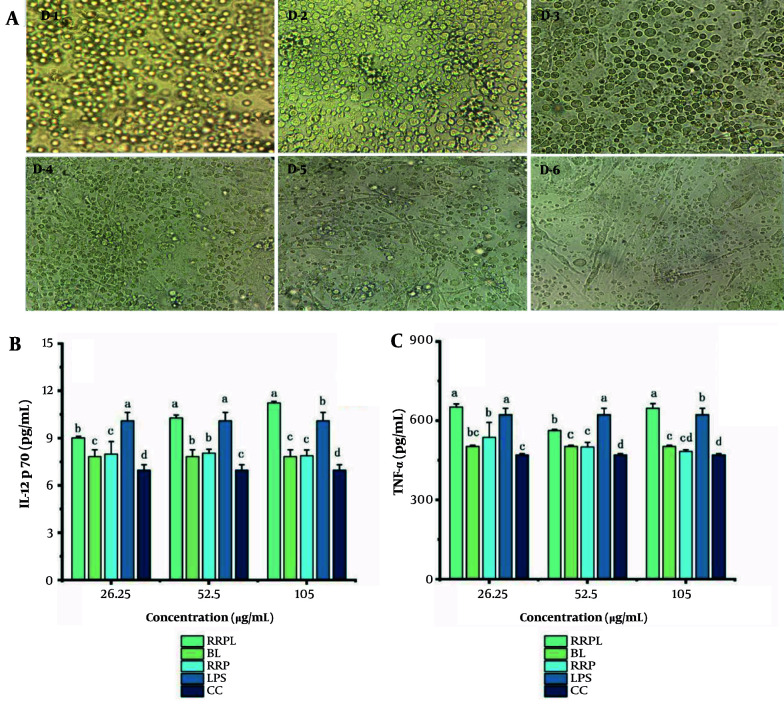
A, morphology of BMDCs during induction and culture, magnified 40 times; B and C, effects of drugs on IL-12p70 and TNF-α in BMDCs (n = 4); the presence of identical letters in superscript a-d indicates no significant difference (P > 0.05) and the different letters in superscript a-d indicates a significant difference (P < 0.05) (Abbreviations: BMDCs, bone marrow-derived dendritic cells; IL-12p70, interleukin-12p70; TNF-α, tumor necrosis factor-α; RRPL, *Rosa rugosa* polysaccharides liposomes; RRP, *R. rugosa* polysaccharides; BL, blank liposomes; CC, cell control).

The in vitro culture of BMDCs is depicted in Figure 10A. On day 1, BMDCs appeared small, round, and uniform in size, growing by closely adhering to the culture vessel wall. By day 6, BMDCs displayed a typical morphology characterized by thick, elongated protrusions extending in all directions, indicating their optimal state for subsequent testing.

The results of IL-12p70 and TNF-α secretion are presented in Figure 10B and C, respectively. The RRPL group at a concentration of 105 μg/mL exhibited a significantly greater ability to enhance IL-12p70 and TNF-α secretion from BMDCs compared with all other groups (P < 0.05). Additionally, at concentrations ranging from 26.25 to 52.5 μg/mL, the RRPL group demonstrated significantly greater promotion of IL-12p70 and TNF-α secretion compared with all other groups, except for the LPS-positive control group (P < 0.05). Moreover, IL-12p70 and TNF-α levels in the RRPL group were significantly higher than those in the RRP group, indicating that RRPL more effectively promote IL-12p70 and TNF-α secretion from BMDCs compared with RRP.

## 5. Discussion

Immune responses are broadly categorized into innate and adaptive immunity. Innate immunity acts as the body’s first line of defense against pathogens, preventing tissue damage (27). This response involves various immune cells, including macrophages, neutrophils, and dendritic cells (DCs). In contrast, adaptive immunity is characterized by its ability to specifically recognize and target pathogens, primarily functioning through peritoneal macrophages (28). Natural polysaccharides derived from plants and animals are increasingly recognized for their immunomodulatory, anti-inflammatory, and antioxidant properties (29). Additionally, they boost immunity by activating various immune pathways and are noted for their excellent biocompatibility and biodegradability, making them suitable for biomedical applications (30).

In a previous report, the polysaccharide isolated from Kushui rose (*R. setate x R. rugosa*) waste exhibited notable immunomodulatory activity by activating the NF-κB signaling pathway (31). In this study, RRP, a crude polysaccharide extracted from *R. rugosa* buds, was utilized as the raw material, and liposomes were employed as the drug carrier, prepared using the reverse-phase evaporation method. Factors investigated included the phospholipid-to-cholesterol mass ratio, drug concentration, and ultrasonication time. Encapsulation efficiency served as the primary evaluation parameter. The optimal formulation for RRPL was identified as a phospholipid-to-cholesterol mass ratio of 6:1, a drug concentration of 1.6 mg/mL, and an ultrasonication time of 8 minutes. Under these conditions, RRPL demonstrated the highest EE of 81.96%, with a corresponding DL of 13.86%.

Li et al. prepared *Portulaca oleracea* L. polysaccharide liposomes using reverse evaporation microporous membrane extrusion, achieving an EE of approximately 38% and a DL capacity of approximately 1% (23). Liu et al. prepared *Polygonatum cyrtonema* flower polysaccharide liposomes using reverse evaporation with an EE of 38% and a DL capacity of 2% (32). Compared with these polysaccharide liposomes, RRPL exhibited a higher EE and DL capacity.

The study found that the maximum safe concentrations on mouse spleen lymphocytes for RRPL and RRP were 210 μg/mL and 105 μg/mL, respectively. This suggests that liposomal encapsulation enhances the safe concentration of RRP while reducing their toxicity. The spleen, the largest secondary lymphoid organ, plays a vital role in both innate and adaptive immune responses (33). It is rich in immune cells such as T and B spleen lymphocytes, whose proliferation is a direct indicator of immunomodulatory activity. In this study, the results showed that RRPL promoted the proliferation of lymphocytes, exhibiting significantly higher levels compared with BL and RRP (P < 0.05). This finding suggests that RRP encapsulated in liposomes further enhance the immune activity of mouse spleen lymphocytes. These results are consistent with those of previous reports (23).

Macrophages, predominantly located in the peritoneal cavity, are essential for both specific and nonspecific immune responses, playing a critical role in defending the body against infections and regulating inflammatory responses. They perform these functions by clearing pathogens and promoting the secretion of inflammatory cytokines such as IL-6, IL-1β, and IFN-γ, which help modulate immune responses (34). In this study, the results demonstrated that RRPL significantly enhanced macrophage phagocytosis, thereby improving their pathogen-clearing ability. Additionally, RRPL markedly increased the secretion of IL-6, IL-1β, and IFN-γ in peritoneal macrophages. In alignment with the findings presented by Liu et al., this study suggests that RRPL can induce immune cell activation by stimulating the proliferation of peritoneal macrophages and enhancing cytokine secretion (22).

The BMDCs, derived from bone marrow stem cells and lymphoid progenitors, are pivotal antigen-presenting cells that activate both innate and adaptive immune responses. In their immature state, BMDCs typically do not secrete cytokines unless stimulated; however, mature BMDCs can produce various cytokines, including IL-12p70 and TNF-α (35). In this study, the results showed that RRPL significantly enhanced the proliferation of BMDCs and increased the secretion of IL-12p70 and TNF-α from induced DCs. These findings align with those documented in the literature, where the study demonstrated that LBPL can significantly increase the secretion of IL-12 and TNF-α (21).

The nano-formulations of natural plant polysaccharides mainly include nanoparticles, nanoemulsions, and liposomes. As a nano-formulation for natural plant polysaccharides, liposomes feature a unique bilayer vesicle structure, enabling them to encapsulate both water-soluble and lipid-soluble components simultaneously, while demonstrating excellent biological membrane compatibility. Their surfaces are easily modified to achieve precise targeted delivery. Through pH-responsive or enzymatic hydrolysis mechanisms, liposomes can effectively control drug release, offering long-acting and low-toxicity characteristics. Compared with nanoparticles and nanoemulsions, liposomes have significant advantages in safety, drug-loading scope, and targeting flexibility, making them highly promising delivery vectors for natural plant polysaccharides.

The administration routes of natural polysaccharide drugs mainly include subcutaneous injection, caudal vein injection, intramuscular injection, and oral administration, with few reports on mucosal administration. However, mucosal administration has advantages such as bypassing the first-pass effect, rapid absorption through rich mucosal blood vessels, convenient administration, and strong patient compliance (36). Tamaddon et al. developed loratadine nanoliposomes using a membrane hydration technique, addressing the challenge of short residence time in the nasal cavity; liposomes demonstrate significant clinical potential for nasal mucosal administration. Therefore, RRPL may also be considered for delivery via the nasal mucosa (37).

### 5.1. Conclusions

The RRPL exhibit a high EE and DL capacity. They show stronger immunological effects on mouse splenic peritoneal macrophages, abdominal macrophages, and BMDCs compared with RRP. These findings suggest that the immunomodulatory activity of RRP can be significantly enhanced through liposome encapsulation. This study provides a theoretical foundation for subsequent in vivo studies in this domain. However, the mechanism for enhancing the immune activity of RRPL will be explored in future studies to compare its potential mechanism of action with RRP.

## Data Availability

The dataset presented in the study is available on request from the corresponding author during submission or after publication.
